# Current alcohol, tobacco, and khat use and associated factors among adults living in Harari regional state, eastern Ethiopia: A community-based cross-sectional study

**DOI:** 10.3389/fpsyt.2022.955371

**Published:** 2022-11-24

**Authors:** Tilahun Bete, Magarsa Lami, Abraham Negash, Addis Eyeberu, Abdi Birhanu, Bekelu Berhanu, Tilahun Abdeta, Shambel Nigussie, Deribe Bekele Dechasa, Kabtamu Gemechu, Dawud Wedaje, Ayichew Alemu, Haregeweyn Kibret, Kefelegn Bayu, Fentahun Meseret, Yideg Abinew, Fenta Wondimneh, Gebisa Dirirsa, Abduro Godana, Jemal Husen, Addisu Alemu, Kabtamu Nigussie, Helina Heluf, Kasahun Bogale, Yadeta Dessie

**Affiliations:** ^1^Department of Psychiatry, School of Nursing and Midwifery, College of Health and Medical Sciences, Haramaya University, Harar, Ethiopia; ^2^School of Nursing and Midwifery, College of Health and Medical Sciences, Haramaya University, Harar, Ethiopia; ^3^College of Health and Medical Sciences, Haramaya University, Harar, Ethiopia; ^4^Department of Clinical Pharmacy, School of Pharmacy, College of Health and Medical Sciences, Haramaya University, Harar, Ethiopia; ^5^School of Medical Laboratory Science, College of Health and Medical Sciences, Haramaya University, Harar, Ethiopia; ^6^Department of Environmental Health, College of Health and Medical Sciences, Haramaya University, Harar, Ethiopia; ^7^School of Public Health, College of Health and Medical Sciences, Haramaya University, Harar, Ethiopia

**Keywords:** psychoactive substances, khat, tobacco, alcohol, associated factor, eastern Ethiopia

## Abstract

**Background:**

Psychoactive substance use becomes a major public health and socioeconomic problem worldwide. Despite its burden and consequences, there is no community-based study conducted on psychoactive substance use and associated factors in eastern Ethiopia. Therefore, this study aimed to assess the magnitude and determinants of current alcohol, tobacco, and khat among adults living in Harari regional state, eastern Ethiopia.

**Methods:**

A community-based cross-sectional study was conducted on 955 adults living in Harari regional state. Participants were randomly recruited using a simple random sampling technique. Data were collected by interviewer-administered structured and semi-structured questionnaires. Data were entered into Epi Data version 3.1 and exported to Stata version 14.0 for analysis. Logistic regression analysis was performed to determine the association between the outcome and independent variables, and the statistical significance was declared at a *p* < 0.5.

**Results:**

Of 955 eligible participants, 95.29% participated in the study. The overall prevalence of current alcohol use, tobacco use, and khat use in this study was 8.24, 14.5, and 63.30%, respectively. The availability of alcohol, being unemployed, and being a current khat user were significantly associated with current alcohol use. Being male, having a low level of education, having peer pressure, having a common mental disorder, being a current alcohol user, and being a khat user were identified as significant predictors for current tobacco use. The age between 31 and 40 years, being a Muslim religion follower, being a farmer, being a current tobacco user, and availability of khat were significantly associated with current khat use.

**Conclusion and recommendations:**

The prevalence of psychoactive substance use in the study area was relatively high compared with that of previous studies. By considering these determinants, screening, early identification, and developing appropriate intervention strategies to prevent and tackle current alcohol, tobacco, and khat use in the community should be of great concern.

## Introduction

A psychoactive substance is a chemical substance that changes the function of the nervous system and results in the alteration in perception, mood, consciousness, cognition, and behavior (1). Globally, more than 35 million people are suffering from severe drug use disorders secondary to psychoactive substance use (2). A total of 11.8 million deaths occurred globally due to psychoactive substance use either directly or indirectly every year, out of which 11.4 million are premature deaths (3). The most common psychoactive substances are khat, alcohol, tobacco, cannabis, and other illicit substances.

In 2016, globally 3.1 billion population use alcohol in the last 12 months and 2.3 billion are current drinkers (3). Around 3 million deaths and 132.6 million disability-adjusted life years every year result from alcohol consumption, which accounts for 5.3% of deaths worldwide, and its use by young people is an increasing concern worldwide (3, 4). Alcohol use disorder is one of the top 20 leading causes of disability worldwide (5). Every year more than 8 million people died due to tobacco smoking and most of those deaths occurred in low- and middle-income countries (5). Globally, the number of people chewing khat is estimated to range from 5 to 10 million, and most of them are in the Horn of Africa and the Arabian Peninsula, especially in Ethiopia, Somalia, and Yemen (6).

Psychoactive substance use has become a major public health and socioeconomic problem worldwide (7). It results in physical, social, and mental health disorders (8, 9). Different studies indicated that psychoactive substance users are at risk to develop cancer, heart diseases, and sexually transmitted diseases including HIV, anxiety disorder, bipolar disorder, and antisocial personality disorder (10–13). Additionally, people who use psychoactive substances regularly face a variety of problems, including scholastic challenges, health-related issues (including mental health), and poor peer interaction (14). There are also impacts on family members, the neighborhood, and the entire society (15).

According to reports by the World Health Organization (WHO), worldwide the prevalence of current alcohol use is 42.29% (3) and tobacco use among males is 36% and among females is 8%. By region in the USA, the prevalence of current alcohol use is 26 and 16%, in Europe 42% and 22%, and in Africa 35% and 7% among males and females, respectively (16). The magnitude of alcohol use, tobacco use, and khat use in Ethiopia ranged in between 13.9 and 23.9 (17, 18), 2.9 and 35.5 (19–22), and 37.8 and 50% (23–25). Based on a national study in Ethiopia, a high prevalence of psychoactive substance use was reported (26).

Ethiopia is one of the countries in which psychoactive substance use is commonly practiced by the community (27). The most commonly practiced psychoactive substance is khat chewing 51% (28). According to a meta-analysis on the prevalence of chewing khat among university students in Ethiopia, the pooled prevalence was 23.22% (29). Regarding alcohol consumption, it ranged from 27 to 31%, and tobacco use was 28% (28, 30–32). According to the 2016 demographic and health survey, 35% of females and 46% of males aged 15–49 years had a history of alcohol consumption in their lifetime in Ethiopia and there was a high consumption rate in urban than in rural areas (33). In 2016, the national prevalence of tobacco use for those aged >15 years was 5% (34). Being male, having stressful live events, peer influence, being single, living in an urban setting, and having conflict with family were factors that were significantly associated with psychoactive substance use in different studies (7, 27, 30, 31).

Despite this burden and consequence, few studies have been conducted on the factors associated with the consumption of psychoactive substances in different parts of Ethiopia (28, 31, 35, 36). Almost all studies that were conducted on psychoactive substance use were conducted only on some groups of people, especially among university students (37–44). A community-based study was not conducted in Harari regional state, eastern Ethiopia, regarding psychoactive substance use. Therefore, this study will have considerable significance. First, it will provide the first information on the prevalence and associated factors. Second, it will contribute valuable data for decision and policymakers, health professionals, and concerned stakeholders who would like to apply some intervention mechanisms regarding the issue. Finally, it will be used as a baseline for future researchers who would like to undertake further investigation on the subject. Hence, this study assessed the magnitude and determinants of alcohol, tobacco, and khat use among Harari region adult residents in eastern Ethiopia.

## Materials and methods

### Study area, design, period, population, and eligibility

A community-based quantitative cross-sectional study was conducted in Harari regional state, eastern Ethiopia from March 1–30, 2022. Harari regional state is one of the 11 regional states in Ethiopia, which is found at a distance of 526 km southeast of the capital city Addis Ababa. The source population of this study was Harari regional state residents. All residents living in randomly selected kebeles with an age >18 years were a study population. Randomly selected households as heads or any other household members >18 years of age and residents who lived more than 6 months in the region and were available during the data collection period were included in the study. People who left their houses for some reasons and were seriously ill during the data collection period were excluded from the study.

### Sample size determination and sampling procedures

The sample size was calculated by using a single population proportion formula with the following statistical assumptions: *n* = the minimum sample size required, p = the estimated proportion of psychoactive substance, z = the standard value of confidence level of alpha (95%), and d = the margin of error between the sample and the population (0.04). For this study, p = 23.7%% [the prevalence from a community-based study conducted in Gondar town (17)].


$$\begin{array}{l} \text{ N}=({\left(\text{Z}\_(\text{α}/2)\right)}^{2}\text{P}\;(1-\text{P}))/{\text{d}}^{2}\\ \text{n}=({\left(1.96\right)}^{2}*0.237(1-0.237))/{\left(0.04\right)}^{2}=\;434. \end{array}$$

**Figure 1:**
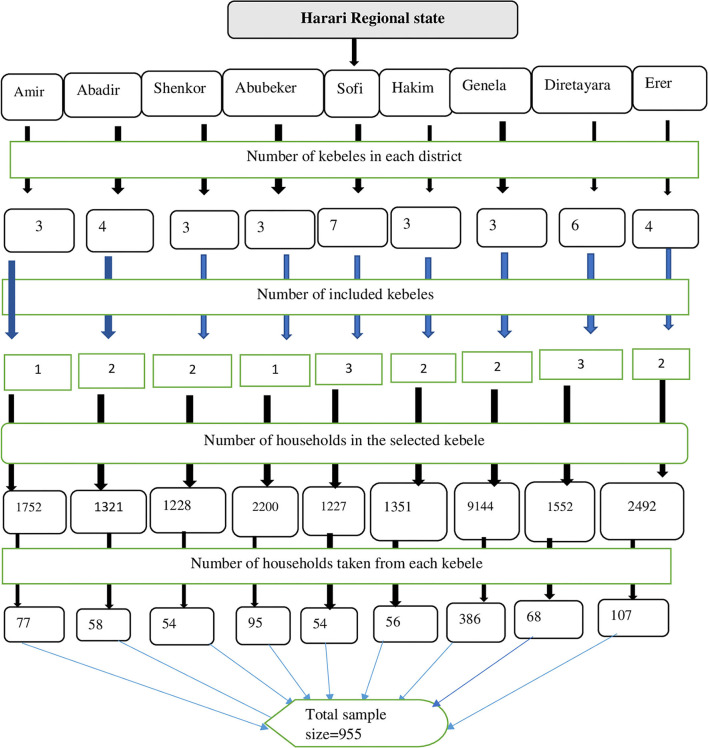
Schematic diagram of the sampling procedures for the study conducted on the prevalence of current psychoactive substance use and associated factors among adults living in Harari regional state, eastern Ethiopia.

Accordingly, with a design effect of 2 and adding a 10% non-response rate, the final sample size was 955. A multi-stage random sampling technique was used to select the study participants. Thirteen kebeles were selected from 9 districts using a simple random sampling (lottery) method. Then, from the selected kebeles, 955 households were allocated proportionally. Each study unit (HH) was selected using a simple random sampling method. At the time when more than one eligible adult was faced in the selected household, a Kish table was used to decide which adult to be interviewed (16) (Figure 1).

### Data collection tools

A structured interview-based questionnaire was used to collect the data. The first part of the questionnaire included questions to assess the sociodemographic characteristics of the participants. The psychoactive substance use was assessed by using Alcohol, Smoking, and Substance Involvement Screening Test (ASSIST). The tool was developed by an international group of substance use researchers of the WHO (45). It is mainly used to assess the psychoactive substance use of lifetime users and current substances (45–47). The common mental disorder was assessed by using a Self-Reporting 20-Item (SRQ-20) questionnaire. The tool was developed by the WHO and assesses primarily non-psychotic mental illness. It has 20 questions that are answered by YES or NO with code “1” which represents the presence of a symptom and code “0” which represents the absence of a symptom. If the respondents' scores are more than six among the 20 questions, they were deemed to have the case, while the remaining were considered not to have the case (48–51).

### Operational definition

#### Common mental disorder

An individual who scored more than six (6) of SRQ-20 questions was considered to have CMD (52).

#### Current substance users

Clients used a specified substance (for non-medical purposes) in the last 3 months (24).

#### Ever substance users

Clients used a specified substance (for non-medical purposes) once in their lifetime (24).

#### Monthly income

According to the World Bank report, individuals with an income of <1.9 $ per day (<2166 ETB per month) are below the poverty line and those with an income of ≥1.9 $ per day (≥2166 ETB per month) are above the poverty line (53).

### Data quality control

A pretest was conducted on 5% of the sample size before data collection in Haramaya town, eastern Ethiopia, to check the clarity, sequence, and applicability of questions and for estimating the time required to collect the data, and necessary corrections were done on the questionnaire based on the finding of the pretest. All data collectors and supervisors were trained for 1 day on the principles, ethics, procedures, and questionnaires in detail. All filled questionnaires were checked for their consistency and completeness.

### Data processing and analysis

Data were entered into Epi Data version 3.1 and exported to Stata version 14.0 for analysis. Descriptive statistical tests were used to provide a clear distribution of the data. Numerical variables were measured as mean and standard deviations, while categorical variables were expressed as frequencies and percentages. Bi-variable logistic regression was conducted to determine the association between independent variables and the outcome variables independently, and those with a *P* < 0.25 and those who have clinical impacts or factors were entered into the final multivariate binary logistic regressions. To measure the strength of the association between independent and outcome variables, OR with their corresponding 95% CI was used. The model fitness was determined by using the Hosmer–Lemeshow test, and all of them were insignificant. A *P*-value of 0.05 was used to declare the presence of statistical significance.

### Ethical consideration

Ethical clearance was taken from the Institutional Health Research Ethics Review Committee (IHRERC) of the College of Health and Medical Sciences of Haramaya University. Then, data collection was initiated after a letter of the corporation was obtained from the Haramaya University, College of Health and Medical Sciences, and submitted to each district and kebele administrators. Official permission was secured from the district and kebele administrators. Additionally, informed voluntary written and signed consent was obtained from all participants after explaining the purpose and importance of the study before the interview. Participation in the study was voluntary, and all the information collected from the participants was kept under the custody of the researcher as confidential. Psychoeducation was given to those individuals who report current psychoactive substance use regarding the impact of substance use on their health. To ensure the safety of data collectors and participants from COVID-19 pandemic disease, training was given to data collectors on the proper use of coronavirus requisition measures.

## Results

### Sociodemographic characteristics of respondents

Of the total 955 eligible participants, 910 participated in the study making the response rate 95.29%. The mean age of the study participants was 42.28 years with a standard deviation of + 12.31 years. Out of the total participants, 508 (55.82%) were female and most of them 733 (80.55%) were urban dwellers. More than three-fourths of 689 (75.71%) of the study participants were Muslims and government employees. The majority of the study participants (*n* = 756, 83.08%) were married and 381 (41.87%) were unable to read and write (Table 1).

**Table 1:** Sociodemographic characteristics of the study participants in Harari regional state, eastern Ethiopia (n = 910).

**Variables**	**Category**	**Frequency (n)**	**Percentage (%)**
Sex	Male	402	44.18
	Female	508	55.82
Age	<30	177	19.45
	31–40	248	27.25
	41–50	187	20.55
	>50	298	32.75
Marital status	Single	82	9.01
	Married	756	83.08
	Divorced	72	7.91
Educational status	No formal education	381	41.87
	Elementary (1–8)	161	17.69
	Secondary	193	21.21
	Diploma and above	175	19.23
Religion	Muslim	689	75.71
	Orthodox	188	20.66
	Protestant	33	3.63
Occupation	Farmer	433	47.58
	Student	55	6.04
	Unemployed	42	4.62
	Governmental employer	380	75.71
Monthly income	Monthly income <2166 ETB	327	35.93
	Monthly income >2166 ETB	583	64.07

### Clinical characteristics of respondents

Of the total respondents, 319 (35.05%) had experienced stressful life events and 178 (19.56%) had common mental disorders.

### The magnitude of substance use among the respondents

The lifetime substance use among the respondents was 641 (70.44%), and the current substance use was 608 (66.81%). The magnitude of current alcohol, tobacco, and khat use among the participants was 75 (8.24%), 132 (14.51%), and 576 (63.30%), respectively.

### Factors associated with current alcohol use

Bi-variable logistic regression was conducted to determine factors associated with current alcohol use among adult residents of Harari regional state, and variables such as sex, religion, educational status, occupational status, khat use, tobacco use, availability of alcohol, peer pressure, and common mental disorder were selected for multivariable logistic analysis for current alcohol use based on clinical factors and *p* < 0.25. However, under multivariate logistic regression, only being unemployed, current khat use, and availability of alcohol were significantly associated with current alcohol use at a *p* < 0.05.

Unemployed individuals were more than three times (AOR = 3.14; 95% CI: 1.12–8.79) more likely to use alcohol than students. Khat users were three times (AOR = 3.14; 95% CI: 1.566.34) more likely to be current alcohol users than non-khat users. The odds of using alcohol were more than two times (AOR = 2.45; 95% CI: 1.34–4.47) more likely among individuals who can easily get alcohol than their counterparts as given in Table 2.

**Table 2:** Factors associated with alcohol use among adult residents of Harari regional state, eastern Ethiopia (*N* = 910).

**Variable**	**Characteristics**	**Alcohol use**		**COR (95%CI)**	**AOR (95%CI)**	***P*-value**
		**Yes**	**No**			
Sex	Female	46	462	1	1	1
	Male	29	373	0.78 (0.48, 1.26)	0.59 (0.34, 1.07)	0.083
Religion	Muslim	31	658	3.68 (1.78, 7.62)	0.46 (0.12, 1.74)	0.253
	Orthodox	41	147	2.78 (0.81, 9.55)	3.45 (0.95, 12.58)	0.061
	Protestant	3	30	1	1	1
Educational status	No formal education	22	359	0.53 (0.29, 1.02)	0.62 (0.29, 1.35)	0.230
	Elementary (1–8)	10	141	1.24 (0.63, 2.43)	1.35 (0.62, 2.95)	0.448
	Secondary	15	178	0.74 (0.36, 1.50)	0.66 (0.30, 1.44)	0.294
	Diploma and above	18	157	1	1	1
Occupational status	Farmer	30	403	0.81 (0.48, 1.36)	0.90 (0.47, 1.72)	0.582
	Student	6	49	1.33 (0.53, 3.35)	1.31 (0.47, 3.62)	0.609
	Unemployed	7	35	2.18 (0 0.89, 5.29)	3.14 (1.12, 8.79)*	**0.029**
	Governmental job	32	348	1	1	1
Khat use	Khat non-user	26	308	1	1	1
	Khat user	49	527	1.10 (0.67, 1.81)	3.14 (1.56, 6.34)*	**0.001**
Tobacco use	Tobacco non-user	55	723	1	1	1
	Tobacco user	20	112	2.35 (1.36, 4.06)	0.99 (0.56, 1.77)	0.979
Availability of alcohol	Not available	53	693	1	1	1
	Available	22	142	2.03 (1.19, 3.44)	2.45 (1.34, 4.47)*	**0.003**
Peer pressure	Not influenced	66	770	1	1	1
	Influenced	9	65	1.62 (0.77, 3.39)	1.81 (0.75, 4.39)	0.188
Common mental disorder	No	52	680	1	1	1
	Yes	23	155	1.94 (1.15, 3.27)	1.63 (0.89, 2.98)	0.111

COR, crude odds ratio; AOR, adjusted odds ratio; CI, confidence interval; 1, Reference *significant at P < 0.05.

### Factors associated with current tobacco use

In the bi-variable logistic regression analysis, variables such as sex, age, religion, educational status, occupational status, monthly income, living place, alcohol use, khat use, availability of tobacco, peer pressure, and common mental disorder were selected based on clinical factors and *p* < 0.25 for multivariable logistic analysis for current tobacco use. However, in multivariate binary logistic regression analysis, no formal education, elementary education, secondary education, alcohol use, khat use, peer pressure, and common mental disorder were significantly associated with tobacco use at a *p* < 0.05.

In this study, the odds of tobacco use among male respondents were about 2.21 times higher than among female participants (AOR = 2.21, 95% CI: 1.43–3.43). The current study also showed that educational status categories of no formal education, elementary education, and secondary education were more than two times more likely to use tobacco compared with the above diploma level of education (AOR = 2.51, 95% CI: 1.13–5.56; AOR = 2.32, 95% CI, 1.02– 5.31; and AOR = 2.89, 95% CI: 1.29–6.48, respectively). Individuals who had peer pressure to use tobacco were three times (AOR = 3.01, 95% CI: 1.68–5.37) more likely to use tobacco than those who had no peer pressure. We also found that individuals who currently use alcohol and khat were more than three times more likely to use tobacco compared with those who did not use (AOR = 3.52, 95% CI: 1.81–6.85, and AOR = 3.48, 95% CI: 1.99–6.05, respectively). The odds of having tobacco use among participants who had common mental disorders were 2.56 times higher as compared to those who had no common mental disorders (AOR = 2.56, 95% CI: 1.56–4.22) (Table 3).

**Table 3:** Factors associated with tobacco use among adult residents of Harari regional state, Ethiopia, 2022 (*N* = 910).

**Variable**	**Characteristics**	**Tobacco use**		**COR (95%CI)**	**AOR (95%CI)**	***P*-value**
		**Yes**	**No**			
Sex	Female	50	458	1	1	1
	Male	82	320	2.35 (1.61, 3.43)	2.21 (1.43, 3.43)*	***P*** **<** **0.001**
Age	<30 years	23	154	0.91 (0.53, 1.57)	0.73 (0.39, 1.35)	0.318
	31–40 years	33	215	0.94 (0.57, 1.53)	0.96 (0.56, 1.65)	0.887
	41–50 years	34	153	1.35 (0.83, 2.22)	1.26 (0.73, 2.19)	0.405
	>50 years	42	256	1	1	1
Religion	Muslim	115	574	3.11 (0.73, 13.16)	1.71 (0.37, 7.91)	0.491
	Orthodox	15	173	1.34 (0.29, 6.17)	0.84 (0.17, 4.26)	0.834
	Protestant	2	31	1	1	1
Educational status	No formal education	71	310	3.41 (1.76, 6.62)	2.51 (1.13, 5.56)*	**0.024**
	Elementary (1–8)	26	135	2.87 (1.37, 6.02)	2.32 (1.02, 5.31)*	**0.045**
	Secondary	24	169	2.12 (1.00, 4.461)	2.89 (1.29, 6.48)*	**0.010**
	Diploma and above	11	164	1	1	1
Occupational status	Farmer	85	350	2.13 (1.41, 3.22)	1.68 (0.96, 2.93)	0.071
	Student	6	49	1.10 (0.44, 2.74)	0.98 (0.36, 2.64)	0.964
	Unemployed	5	37	1.22 (0.45, 3.28)	1.48 (0.50, 4.37)	0.473
	Governmental job	38	342	1	1	1
Monthly income	<2,166	60	267	1.59 (1.09, 2.32)	0.79 (0.82, 1.97)	0.280
	>2,166	72	511	1	1	1
Living place	Rural	49	128	2.99 (2.01, 4.48)	1.45 (0.79, 2.66)	0.233
	Urban	83	650	1	1	1
Alcohol use	Alcohol non-user	112	723	1	1	1
	Alcohol user	20	55	2.35 (1.36, 4.07)	3.52 (1.81, 6.85)*	***P*** **<** **001**
Khat use	Khat non-user	19	315	1	1	1
	Khat user	113	463	4.05 (2.44, 6.72)	3.48 (1.99, 6.05)*	***P*** **<** **001**
Availability	Not available	95	651	1	1	1
	Available	37	127	1.99 (1.31, 3.05)	1.14 (0.69, 1.87)	0.600
Peer pressure	Not influenced	101	735	1	1	1
	Influenced	31	43	5.25 (3.16, 8.71)	3.01 (1.68, 5.37)*	***P*** **<** **0.001**
Common mental disorder	No	91	641	1	1	1
	Yes	41	137	2.11 (1.39, 3.18)	2.56 (1.56, 4.22)*	***P*** **<** **0.001**

COR, crude odds ratio; AOR, adjusted odds ratio; CI, confidence interval; 1, Reference *significant at P < 0.05.

### Factors associated with khat use

In the bi-variable logistic regression, variables such as sex, age, religion, educational status, occupational status, living place, alcohol use, tobacco use, availability of khat, peer pressure, and common mental disorder were selected based on clinical factors and *p* < 0.25 for multivariable logistic regression for the outcome variable khat use. In the final multivariate logistic regression, being Muslim, farmer, tobacco use, and availability of khat were significantly associated with the outcome variable at a *p* < 0.05.

Muslim religion followers were more than three times (AOR = 3.38, 95% CI: 1.53–7.49) more likely to use khat than followers of the protestant religion. In the current study, we found that individuals who had easy access to khat were more than five times (AOR = 5.41, 95% CI: 3.23–9.05) more likely to use khat than those who did not have easy access. We also found that tobacco users were about three times (AOR = 3.58, 95% CI: 2.07–6.18) more likely to use khat compared with those who did not use it. The odds of using khat were 1.62 times (AOR = 1.16, 95% CI: 1.11–2.35) more likely to occur among farmers than governmental employees. The age between 31 and 40 years was a protective factor that participants who were in the age group of 31–40 years were 0.67 times (AOR = 0.67, 95% CI: 0.46–0.97) less likely to use khat than those who were aged >50 years (Table 4).

**Table 4:** Factors associated with khat use among adult residents of Harari regional state, Ethiopia, 2022 (*N* = 910).

**Variable**	**Characteristics**	**Khat use**		**COR**	**AOR (CI)**	***P*-value**
		**Yes**	**No**			
Sex	Female	256	146	1	1	1
	Male	320	188	1.03 (0.79, 1.35)	0.89 (0.65, 1.22)	0.461
Age	<30 years	115	32	0.97 (0.65, 1.43)	0.92 (0.60, 1.40)	0.697
	31–40 years	143	105	0.71 (0.5, 1.00)	0.67 (0.46, 0.97)*	**0.036**
	41–50 years	122	62	0.98 (0.66, 1.43)	0.88 (0.58, 1.33)	0.531
	>50 years	196	102	1	1	1
Religion	Muslim	467	222	3.68 (1.78, 7.62)	3.38 (1.53, 7.49)*	**0.003**
	Orthodox	97	91	1.87 (0.87, 4.01)	1.92 (0.84, 4.41)	0.122
	Protestant	12	21	1	1	1
Educational status	No formal education	244	137	1.27 (0.88, 1.84)	0.73 (0.46, 1.14)	0.166
	Elementary (1–8)	116	45	1.84 (1.17, 2.91)	1.45 (0.88, 2.38)	0.144
	Secondary	114	79	1.03 (0.68, 1.56)	0.89 (0.57, 1.41)	0.644
	Diploma and above	102	73	1	1	1
Occupational status	Farmer	302	131	1.77 (1.33, 2.36)	1.62 (1.11, 2.35)*	**0.012**
	Student	37	18	1.58 (0.87, 2.87)	1.51 (0.79, 2.87)	0.205
	Unemployed	22	20	0.84 (0.45, 1.59)	0.81 (0.41, 1.62)	0.556
	Governmental job	215	165	1	1	1
Living place	Rural	132	45	1.91 (1.32, 2.76)	0.91(0.56, 1.48)	0.716
	Urban	444	289	1	1	1
Alcohol use	Alcohol non-user	527	308	1	1	1
	Alcohol user	49	26	1.10 (0.67, 1.81)	0.93 (0.52, 1.66)	0.795
Tobacco use	Tobacco non-user	463	315	1	1	1
	Tobacco user	113	19	4.05 (2.44, 6.72)	3.58 (2.07, 6.18)*	***P*** **<** **0.001**
Availability	Not available	432	314	1	1	1
	Available	144	20	5.23 (3.21, 8.54)	5.41 (3.23, 9.05)*	***P*** **<** **0.001**
Peer pressure	Not influenced	518	318	1	1	1
	Influenced	58	16	2.23 (1.26, 3.94)	1.66 (0.87, 3.17)	0.121
Common mental disorder	No	472	260	1	1	1
	Yes	104	74	0.77 (0.55, 1.08)	0.78 (0.53, 1.14)	0.200

COR, crude odds ratio; AOR, adjusted odds ratio; CI, confidence interval; 1, Reference *significant at P < 0.05.

## Discussion

This study aimed to assess the magnitude and factors associated with current alcohol, tobacco, and khat use among Harari regional state adult residents. The overall prevalence of current psychoactive substance use was 66.8%. The magnitude of current alcohol, tobacco, and khat use in this study was 8.24, 14.51, and 63.30%, respectively. This study was self-reported substance use that undermines the prevalence and it is a cross-sectional study design, which cannot allow establishing a temporal relationship between current alcohol use, tobacco use, and khat use, with associated factors, which were the limitations of the study, whereas a community-based study with a sufficient sample size could be seen as the strength of this study.

The magnitude of current alcohol use in this study was 8.24%. The finding is lower than studies conducted in Ethiopia 13.9% (18), Gondar 23.7% (17), Mekelle 25.1% (43), South Ethiopia 22.4% (54), Kenya 12.8% (55), South Africa 20% (56), and Nigeria 66.7% (57). The possible reason for the discrepancy might be due to the tools used to assess the current alcohol use. This study used ASSIST (Alcohol, Smoking, and Substance Involvement Screening Test), whereas the studies conducted in South Ethiopia and Gondar used AUDIT, and they assessed based on the 12-month duration. The other possible reasons might be due to the study participant's religion in this study; most of the participants (75.71%) were Muslims who prohibited the use of alcohol in their religious doctrine. Additionally, cultural and social living style differences across the communities might be one of the reasons for the variation. On the contrary, the finding of this study is higher than the studies conducted in Addis Ababa, Ethiopia, which was 2.7% (58) and Comoros 1.3% (59). This discrepancy might be related to tool difference, the study done in Addis Ababa used Fast Alcohol Screening Test (FAST) and AUDIT to assess the alcohol use, and focused on identifying hazardous alcohol users which makes lower than this study finding.

The availability of alcohol is significantly associated with current alcohol use. This agrees with previous studies of South Africa (60) and Africa (61). This is might be because if alcohol is easily available at a relatively low price and also locally produced at any time for those who want to use it, it leads to increased use of alcohol.

Being unemployed is significantly associated with current alcohol use. This result was in line with the Ethiopian EDHS study (62). This is might be because those who have no job may feel stressed and depressed and they want to hide and want to spend their time using alcohol as a defense mechanism. The other possible reason might be that being unemployed may precipitate or relapse into alcohol use. Current khat use was also associated with current alcohol use in this study. This agrees with a study conducted in Addis Ababa, Ethiopia (35). This might be due to that most khat chewers consume alcohol in parallel, and it is a common practice to use alcohol after chewing Khat to neutralize its effect. The other reason might be that khat is a gateway to other substances (35, 38, 63, 64).

The prevalence of current tobacco use in this study was 14.51%, which is in line with studies conducted in Mekelle, Ethiopia, 11.4% (43), South Africa 15% (65), and Kenya 13.5% (66), but lower than studies from eastern Ethiopia 28% (30), Arba Minch, Ethiopia, 20.5% (19), Jimma, Ethiopia 35.5% (20), South Africa 17.6% (21), Nigeria 20.6% (67), and Tanzania 24% (68). The possible reason for the discrepancy might be that the study which was conducted in eastern Ethiopia was conducted in the rural communities, whereas this study included both the rural and urban communities. The other reason may be the variation of the tools used to assess tobacco use, and this study used ASSIST, whereas the Arba Minch study used WHO STEP wise, and eastern Ethiopia used the Global Tobacco Surveillance System (GTSS). On the contrary, the finding of this study is higher than the studies on EDHS where the prevalence was 4% (69), Ethiopia 3% (70), and Addis Ababa 2.9% (71). The possible reason for the discrepancy might be that the EDHS survey was conducted throughout the country, whereas this study included specific areas and the community of Addis Ababa might have better educational status and awareness of the impact of tobacco than Harari regional state community.

The odds to use tobacco are more than two times more likely to occur among males than females. This is in line with studies conducted in sub-Saharan Africa and lower–middle-income countries (22, 66). The possible reason for this might be that tobacco industries frequently portray their product as it has advantageous for sexual activity and masculine activity (72). In addition to this, the perception of the community favors smoking for males over females, and females are more socially stigmatized and ashamed if they smoke (72–74).

The current study also showed that educational status categories with a low level of education, unable to read and write, and elementary and secondary levels of education were more than two times more likely to use tobacco as compared to the above diploma level of education. This finding agrees with previous studies (75). The low educational level might lead to a low level of knowledge or awareness regarding the impact of cigarette smoking, and maybe they are easily influenced by peer pressure (76, 77).

In the current study, we found that individuals who had peer pressure to use tobacco were 3.01 times more likely to use tobacco than those who had no peer pressure. Peer pressure either directly or indirectly leads individuals to substance use. Directly through offering, inviting, or encouraging influences to smoke cigarettes, indirect peer pressure can occur through changing the negative perception toward tobacco as normative and making the environment more conducive to using tobacco. In addition to this, some individuals explain it as modernization and a role model (78, 79).

The study also found that individuals who are currently using alcohol and khat were about three times and nearly two times more likely to use tobacco, respectively, as compared to those individuals who did not use it. This agrees with studies from Ethiopia EDHS (62). This might be due to that after the person gets intoxicated to get more pleasure they may use cigarette. The other reason might be that individuals may use alcohol and tobacco concomitantly (80).

The odds of using tobacco are more than two times more likely to occur among individuals who had common mental disorders than their counterparts. This agrees with the finding of other studies in South Africa (81). This might be due to that a person who is living with a common mental disorder might use tobacco as self-medication to relieve their emotional disturbance, especially from depressive symptoms who have a negative attitude toward themselves and the future, low self-esteem, and loss of interest (82). The other reason might be comorbidity with depressive symptoms and severe forms of nicotine withdrawal symptoms (83, 84).

The magnitude of current khat use in this study was 63.30%. This finding is higher than that of previous studies including Ethiopia EDHS 2016 8.4% (85), 17.20% (44), Butajira 50% (23) Jimma 37.8% (52), Mekelle 9.2% (43), Hosanna town 58.0% (36), and Kenya 36.8% (86). The possible reason might be that the eastern part of Ethiopia is the origin of khat and the most popular for khat production and use (87). Khat use in this study area is one of the culturally accepted practices and the primary source of economic income for the community. On the other hand, this finding is lower than the finding of Jimma which was 68% (88). The main reason for this discrepancy might be that the finding of Jimma is a lifetime substance use, whereas this study is current substance use or within 3-month duration. The other reason might be that the Jimma study was taken place in the rural district, but this study was conducted in both the urban and rural districts.

The study found that being a Muslim religion follower was significantly associated with khat use. This was in line with previous Ethiopian studies in Jimma (52), Agaro (89), Hosanna town (36), and Butajira (63). The possible reason might be that the perception of the community in the use of khat has been confined to the Muslim religion followers and culturally accepted by the community and passed from generation to generation. The other reason might be that in this study there are a high number of Muslim religion followers compared with other religions. Thus, this might result in a variation (90).

The study also revealed that being a farmer in their occupation and availability were significantly associated with khat use. This might be that as khat is the main source of economic income for the farmer in the study area, cultivating it easier leads them to utilize it more. Regarding availability, it is available everywhere at a low cost and even it is cultivated on the farm of rural farm areas as it is their main economic income compared with other areas.

Current tobacco use was also identified as one predictor of current khat use. This is in line with a study conducted in Yemen (91) in which 70% of the tobacco users use khat before tobacco use. The other reason might be that those who use khat also use tobacco at the same time for pleasure. The age between 31 and 40 years was found to be significantly associated with khat use. Middle-aged groups who represent the most productive sections of society are most affected by the khat chewing habit and conform to the society's culture (63).

## Conclusion and recommendations

The finding of this study revealed that alcohol, tobacco, and khat were commonly practiced substances use among Harari residents. Availability of substance were associated with current alcohol and khat use whereas, presence of common mental disorder and peer pressure were associated with current tobacco use. So, early screening and identification and development strategies to prevent and tackle current alcohol use, tobacco use, and khat use have paramount significance. Governmental and non-governmental organizations are better to emphasize the identified factors and provide psychosocial support to the residents. Further studies should be conducted by including a comparative and longitudinal study to verify the causal relationship between correlates and current alcohol use, tobacco use, and khat use.

## Data availability statement

The original contributions presented in the study are included in the article/supplementary material, further inquiries can be directed to the corresponding author.

## Ethics statement

The studies involving human participants were reviewed and approved by the Institutional Health Research Ethics Review Committee (IHRERC) of the College of Health and Medical Sciences of Haramaya University. The patients/participants provided their written informed consent to participate in this study.

## Author contributions

TB, TA, DW, KG, AE, KN, KB, and HH participated in proposal writing, analysis, discussion, and preparing the manuscript. AB, HK, AA, KB, YD, DBC, TA, ML, SN, AN, GD, BB, JH, KN, and AG are participated in organizing, data cleaning, and supervising data collection process. All authors participated in preparing and approving the manuscript.

## Funding

The whole required (materials and humanitarian) cost for this research work was covered by the Haramaya University.

## Conflict of interest

The authors declare that the research was conducted in the absence of any commercial or financial relationships that could be construed as a potential conflict of interest.

## Publisher's note

All claims expressed in this article are solely those of the authors and do not necessarily represent those of their affiliated organizations, or those of the publisher, the editors and the reviewers. Any product that may be evaluated in this article, or claim that may be made by its manufacturer, is not guaranteed or endorsed by the publisher.

## Abbreviations

AOR: Adjusted Odd Ratio
ASSIST, Alcohol, Smoking: and Substance Involvement Screening Test
CMD: Common Mental Disorder
COR: Crude Odd Ratio
GOVT: Government
KM: Kilometer
NGO: Non-Governmental Organization
OR: Odds Ratio
PI: Principal Investigator
USA: United States of America
WHO: World Health Organization.
